# Exploring the experiences of cognitive symptoms in Long COVID: a mixed-methods study in the UK

**DOI:** 10.1136/bmjopen-2024-084999

**Published:** 2025-01-25

**Authors:** Amy Miller, Ning Song, Manoj Sivan, Rumana Chowdhury, Melanie Rose Burke

**Affiliations:** 1 University of Leeds, Leeds, UK; 2 Leeds Teaching Hospitals NHS Trust, Leeds, UK

**Keywords:** COVID-19, QUALITATIVE RESEARCH, PUBLIC HEALTH, Cognition

## Abstract

**Abstract:**

**Objective:**

To explore the lived experiences and extent of cognitive symptoms in Long COVID (LC) in a UK-based sample.

**Design:**

This study implemented a mixed-methods design. Eight focus groups were conducted to collect qualitative data, and the Framework Analysis was used to reveal the experiences and impact of cognitive symptoms. A self-report questionnaire was used to collect the quantitative data to assess the perceived change and extent of symptomology post COVID-19.

**Setting:**

Focus groups were conducted in April 2023 online via Zoom and in-person at the University of Leeds, UK.

**Participants:**

25 people with LC living in the UK participated in the study. Participants were aged 19–76 years (M=43.6 years, SD=14.7) and included 17 women and 8 men.

**Results:**

Reduced cognitive ability was among the most prevalent symptoms reported by the study participants. Three key themes were identified from the qualitative data: (1) rich accounts of cognitive symptoms; (2) the impact on physical function and psychological well-being and (3) symptom management. Descriptions of cognitive symptoms included impairments in memory, attention, language, executive function and processing speed. Cognitive symptoms had a profound impact on physical functioning and psychological well-being, including reduced ability to work and complete activities of daily living. Strategies used for symptom management varied in effectiveness.

**Conclusion:**

Cognitive dysfunction in LC appears to be exacerbated by vicious cycle of withdrawal from daily life including loss of employment, physical inactivity and social isolation driving low mood, anxiety and poor cognitive functioning. Previous evidence has revealed the anatomical and physiological biomarkers in the brain affecting cognition in LC. To synthesise these contributing factors, we propose the Long-COVID Interacting Network of factors affecting Cognitive Symptoms. This framework is designed to inform clinicians and researchers to take a comprehensive approach towards LC rehabilitation, targeting the neural, individual and lifestyle factors.

**STRENGTHS AND LIMITATIONS OF THIS STUDY**

- This study used a mixed-methods approach, combining qualitative and quantitative methods of data collection to capture the experiences of Long COVID (LC) and the severity of cognitive dysfunction among other symptoms in LC.
- The research team was interdisciplinary and interprofessional, combining expertise from LC rehabilitation, neurology, cognitive psychology and qualitative methods.
- This study may be limited by a self-selecting, potentially biased sample consisting of mostly female, middle-aged participants.
- The involvement of multiple facilitators may have led to different areas of focus during the discussions.

## Introduction

 Postacute COVID-19 syndrome, also known as Long COVID (LC), is the continuation or development of new symptoms 3 months after the initial SARS-CoV-2 infection, with symptoms lasting for at least 2 months.^1^ In 2024, an estimated 2 million people living in private households in the UK (3.3% of the population) were experiencing self-reported LC^2^ with common symptoms including fatigue, breathlessness, muscle ache, general malaise and cognitive dysfunction.^3^ The Office for National Statistics^2^ reported that the second most common symptom following fatigue was difficulty concentrating, reported by 52% of respondents. Furthermore, cognitive dysfunction was found to be one of the most common symptoms in a multinational survey of 3762 people with LC, reported in ~88% of individuals.^4^

Neuropsychological assessments and subjective reports have shown cognitive impairments in memory, attention, executive function and verbal fluency following SARS-CoV-2 infection in comparison to individuals without infection.^5 6^ Objectively measured memory deficits following COVID-19 infection have been found to increase in severity, in line with the severity of self-reported ongoing symptoms.^7^ A UK Biobank study found poorer cognitive accuracy in working memory, attention, reasoning and motor control in those who had been infected with SARS-CoV-2 than controls, with the largest deficits found in individuals with ≥12 weeks of symptoms, continuing to 2 years in some individuals.^8^ Persistent cognitive impairment in LC is associated with anatomical and physiological biomarkers. Magnetic resonance images and objective cognitive tests recorded before and after SARS-CoV-2 infection have revealed reduced grey matter in the orbitofrontal cortex and parahippocampal gyrus and reduced global brain size correlated with impaired cognitive performance in those who had been infected than controls.^9^ On a cellular level, SARS-COV-2 infection has been linked to tau pathology in the brain,^10^ and the viral sequence is capable of coding for cytotoxic amyloid proteins with comparable toxicity to proteins found in Alzheimer’s disease.^11^ Further biomarkers associated with LC include chronic neuroinflammation associated with a cascade of neurotoxic events^12 13^ and hypoxia.^14 15^ Collectively, these factors contribute to accelerated cognitive ageing and a heightened risk of dementia in individuals with LC.^16^

Previous qualitative studies have reported the profound, negative impact of LC on daily functioning and quality of life including high prevalence of mental health problems, change or loss of occupational status, difficulty accessing and navigating healthcare services, feelings of isolation, stigma, shame and loss of personal identity.^17^ One previous qualitative study has reported the experiences of ‘brain fog’ in COVID-19 long haulers,^18^ a colloquial term describing a state of confusion, forgetfulness and slowing of mental abilities.^19 20^ However, focus group discussions conducted by Callan *et al*^18^ were held early in the COVID-19 pandemic in 2020, and to our knowledge no further studies have directly investigated the qualitative experiences of cognitive symptoms in LC. Given the heightened risk of neurodegeneration and dementia,^16^ and the detrimental impact on daily life associated with LC,^17^ it is crucial to have a current understanding of the impact of cognitive impairment on the lives of people with LC. By combining qualitative and quantitative methods, this study aimed to capture both in-depth experiences and quantify the extent of cognitive symptoms. This mixed-methods approach was used to enhance the validity and triangulation of the research, to inform a comprehensive understanding of cognitive symptoms in LC. In this study, people with LC living in the UK took part in focus group discussions and a Post-Long COVID-19 Questionnaire. The following questions were explored: (1) What is the extent of cognitive symptoms in LC? (2) What is the lived experience of cognitive symptoms in LC?

## Methods

### Study design

The study implemented a mixed-methods design to explore the experiences of cognitive symptoms in LC. Eight exploratory focus groups were conducted in April 2023 to collect the qualitative data. Four of the groups were held in person at the School of Psychology, University of Leeds (UK), and four were held online via Zoom. The online discussions were held to make the study more accessible, allowing participation from individuals living outside the region or those with mobility impairments. Participants completed a Post-Long COVID-19 Questionnaire, detailed below, providing a quantitative measure of symptom prevalence and severity. A mixed-methods approach was employed to capture both the quantitative nature of symptoms and the qualitative insights into the subjective impacts on daily life. This combined approach provides a comprehensive understanding of the complex nature of cognitive symptoms and their effects.

### Participants

Participants included 25 adults with LC. Eligibility criteria included individuals with self-reported LC who lived in the UK. One participant did not return the Post-Long COVID-19 Questionnaire so were removed from the analysis. The final sample size of 24 participants was sufficient to reach data saturation, where no additional codes or insights emerged from further discussions, ensuring the themes were rich and fully addressed the research question. Participants were recruited via social media advertisement on Facebook, Instagram, LinkedIn and NextDoor, via advertisement in the Chronic Pain & Fatigue Network at the University of Leeds and the English National Opera Breathe Programme for LC, and by word of mouth. Participants were reimbursed with a £20 Love2Shop voucher.

### Quantitative data collection

The Post-Long COVID-19 Questionnaire was adapted from previous NHS questionnaires measuring symptoms in LC (available at https://ardenhousemedicalpractice.co.uk/navigator/post-long-covid-19-syndrome-questionnaire/) in the validated Patient Health Questionnaire-9 format. This questionnaire measured participants’ perceived health in: breathing, mobility, energy, mood, mental ability and physical health^21^ (see online supplemental materials). The questionnaire assessed the severity of symptoms using a mixture of binary questions with ‘yes’ or ‘no’ responses, and scaled questions in which the rating scale comprised: (0) normal, (1) mild, (2) moderate and (3) severe. Participants rated their self-perceived change in symptom severity on 0–10 point Likert scales before and after they contracted LC.

### Qualitative data collection

The focus group discussions were audio-recorded and lasted approximately 2 hours. Participants were informed that the discussions were recorded and that any information they gave was confidential and would be anonymised. There were eight groups each containing two to five participants and one of four facilitators. These groups’ sizes were selected as previous evidence has shown that a larger number of groups containing fewer participants can yield more information than fewer groups with more participants per group.^22^ Facilitators informally followed guidance questions to encourage discussions around the main symptoms and experiences (see box 1). The guided questions were intentionally broad to encourage participants to explore various aspects of their experiences with LC. We intended to refine the analysis from this broader discussion to focus on key topics of particular relevance and importance. The questions were used to guide the sessions while the content of the discussions was self-directed by the participants in the focus groups.

Box 1 **Guided discussion questions followed by facilitators**

**Guided discussion questions**

- What symptoms post-COVID-19 are most distressing for you?
- What can you not do now that you could do before LC?
- How has LC affected your quality of life?
- What can clinical services provide to help with your everyday functioning?
- What services can society provide to help with your symptoms?
- What self-help strategies have worked for you?
- What research would most help people with LC get back to normal functioning?

The questions were intended to guide broad discussions around the experiences of Long COVID (LC). The analysis focused on key themes of relevance and importance, in this case, the experiences of cognitive symptoms.

### Data management and analysis

First, audio recordings were transcribed verbatim and anonymised. To maintain confidentiality, personal identifiers were removed from the data, and access was restricted to the individuals directly involved in the data analysis. Participants’ names were replaced with pseudonyms for anonymity. The data were analysed according to the Framework Analysis proposed by Ritchie and Spencer.^23^ The Framework Analysis provides a structured yet flexible approach to analysing qualitative data, facilitating the systematic organisation of large datasets and diverse participant experiences into themes. The Framework Analysis was selected over other qualitative methods, such as grounded theory and interpretative phenomenological analysis, as the focus of this study was not to generate new theory or deeply analyse lived experiences but to explore and categorise experiences within a broader thematic framework.

The Framework Analysis involved five stages: (1) the process of data familiarisation involved repeatedly reading the transcripts and listening to the audio recordings, which allowed for the identification of initial codes; (2) data were ‘open coded’ and a framework was developed and refined within the research team; (3) manual coding was performed in NVivo (R14.23.1, QSR International) to index the data according to the framework. A second analyst (SM) independently double-coded 25% of the data (2/8 transcripts) to assess the quality of the coding and to enhance the reliability and validity of the data analysis. Any differences in the coding were resolved by consensus and the framework was altered accordingly. Then, the framework was applied to all remaining transcripts. To ensure consistency in the coding and reduce possible bias, the second analyst (SM) checked 20% of the coding. Data saturation was reached when no new codes emerged from the analysis. The initial framework was developed from the broader discussions and from this framework we identified common codes which were relevant to the research question to develop into themes. (4) In the charting stage, the coded data were arranged in a system to organise the data into relevant themes. (5) Theme development involved mapping and interpreting codes, identifying relationships between them, and systematically grouping them into subthemes. These subthemes were then further refined and categorised into overarching themes that captured the key patterns and insights from the data. All stages of the data analysis, framework and theme development were reviewed by an interdisciplinary research team, combining expertise from cognitive psychology and qualitative methods. Discussions within the research team were held to inform the process. While this analysis focused on investigating the experiences of cognitive symptoms, we have also reported on the broader perceived ‘needs’ of people with LC in our previous publication of this study.^24^

### Patient and public involvement

Participants were invited to review the manuscript prior to submission to ensure the findings accurately represented their views and experiences.

## Results

Participants were aged 19–76 years (M=43.6 years, SD=14.7; 17 women and 8 men). Participants were eligible if they had self-reported LC and lived in the UK. 92% (22/24) of participants had a clinical diagnosis of LC. The duration of symptoms experienced varied between 4 months and 2.8 years (M=77.96 weeks, SD=32.58). 21 participants had British nationality, 1 had French, 1 had Finnish and 1 had Chinese nationality.

### Quantitative results

**Figure 1:**
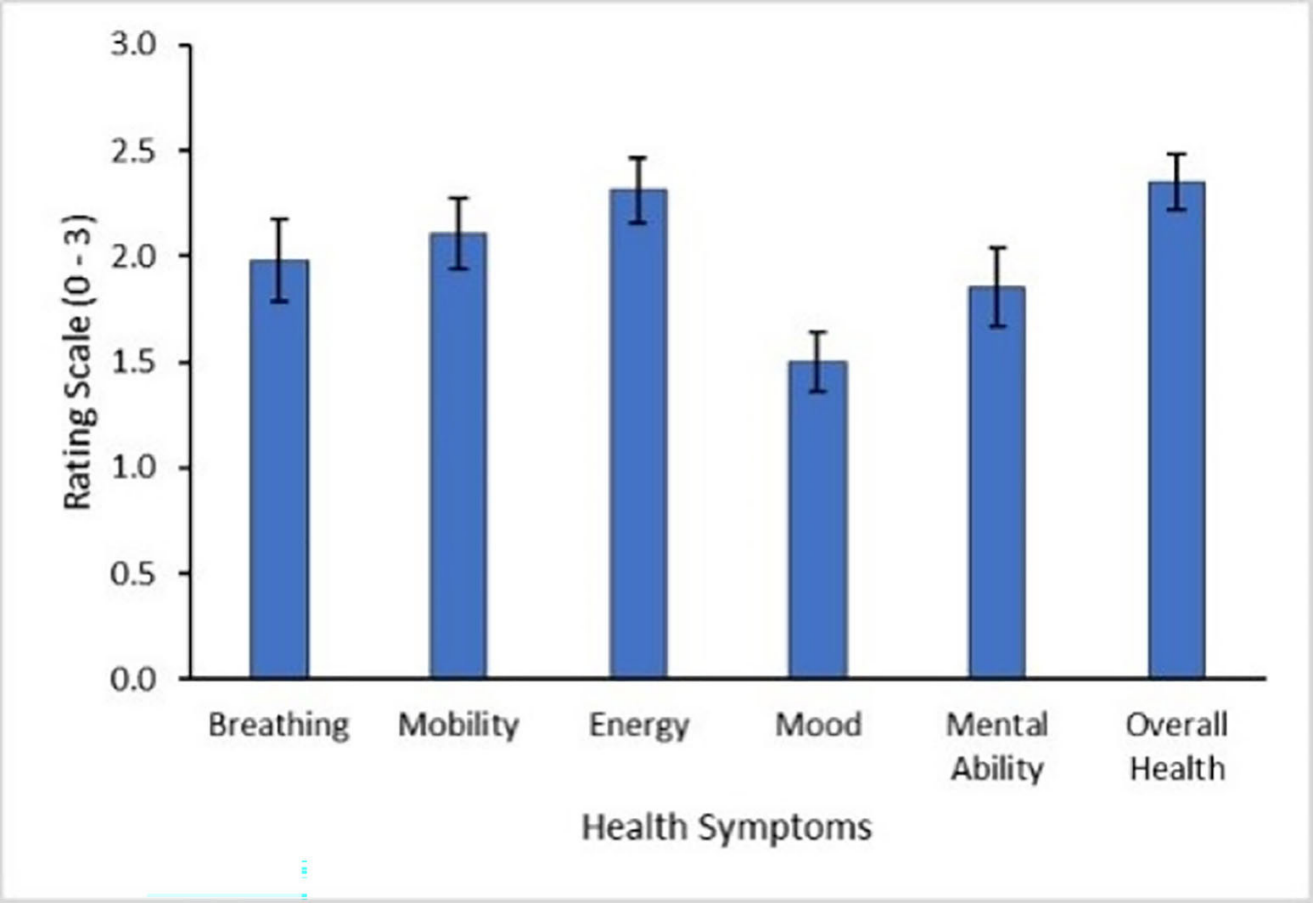
Participant self-reported ratings of symptom severity across health symptoms. High scores indicate greater severity of symptoms. (0) normal, (1) mild, (2) moderate and (3) severe. Error bars show SEM. N=24.

Results from the Post-Long COVID-19 Questionnaire showed that participants experienced wide-ranging symptoms following the development of LC, see figures1  2. The most severe symptoms, with the greatest change after disease contraction, were decreases in energy, mobility, breathing and mental ability.

**Figure 2:**
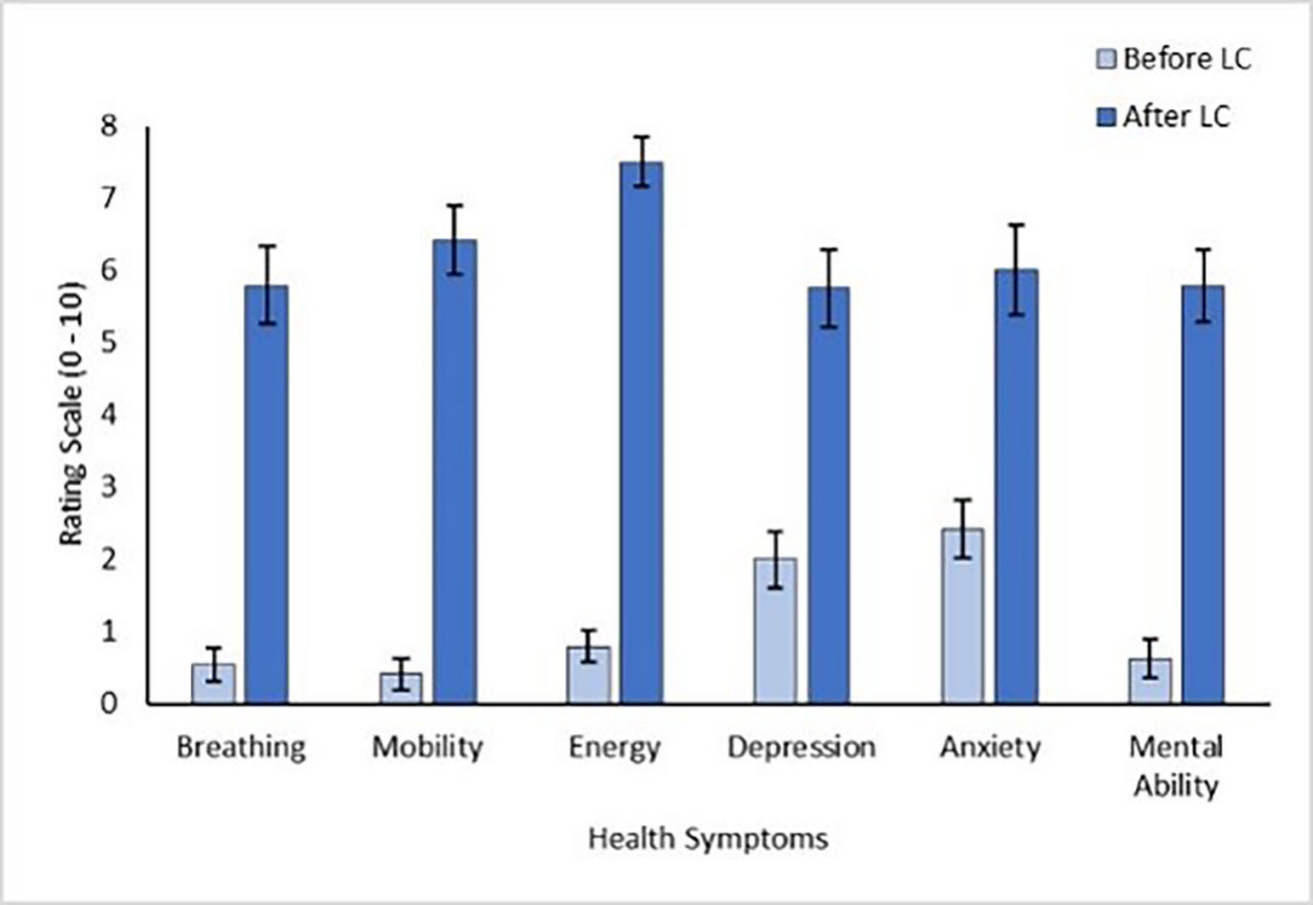
Participant self-reported symptom change before and after contracting Long COVID (LC). High scores indicate greater symptom severity. Error bars show SEM. N=24.

In terms of cognition, 87.50% (21/24) of participants reported that their ability to concentrate had worsened, 83.33% (20/24) reported that their short-term memory had declined and 75.00% (18/24) reported that they or their family had identified a difference in the way they communicated. Participants reported a 10-fold increase in concern about their cognition following LC (M=5.83, SEM=0.61) compared with before LC (M=0.54, SEM=0.27).

Almost all participants reported that their illness and cognition affected their ability to work (95.65%; 22/23; 1 participant was retired). All participants (24/24) requested to be informed of future LC research opportunities showing clear motivation to advance understanding and treatment for persistent symptoms.

### Qualitative results

**Figure 3:**
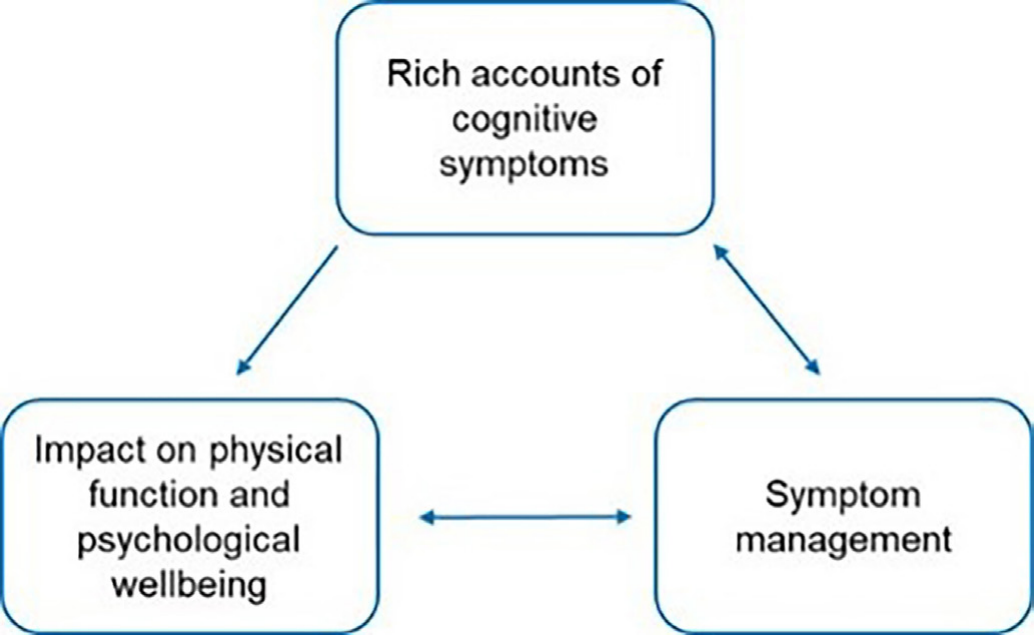
A map of the relationships between themes.

Data from the focus group discussions were categorised into three interconnected themes, see figure 3. Theme 1: rich accounts of cognitive symptoms, theme 2: impact on physical function and psychological well-being and theme 3: symptom management. Participants experienced various cognitive impairments (theme 1) which impacted daily functioning and psychological well-being (theme 2). Participants attempted to manage the symptoms and impact on their daily lives (themes 1 and 2) using strategies (theme 3). Oftentimes these strategies (theme 3) were tedious to implement or ineffective, further exacerbating the impact on physical and psychological functioning (theme 2). See theme descriptions for further details.

### Theme 1: rich accounts of cognitive symptoms

The majority of participants (20/24) described experiences of cognitive symptoms associated with LC. The most commonly reported symptoms were difficulties with memory and language, followed by attention, executive function and reduced processing speed. Participants frequently used the term ‘brain fog’ to describe their symptoms.

#### Memory

Participants described experiences of memory deficits which were consistent with definitions of working memory, long-term memory (declarative and non-declarative memory) and prospective memory. Participants were concerned about the consequences of these memory impairments on their safety. For example, Participant 1 was forgetting to take their medication and reported “I could be overdosing and I’ve no idea.” Other participants were worried about the impact of their memory difficulties on their perceived image, as Participant 18 described “it makes you look stupid” after missing appointments. Participants described a need to review information multiple times before they could process, retain and remember it:

I'll go back to the book and think I don't remember this bit at all and be going back a chapter… not necessarily reading it in detail, but scanning through and it will kind of prompt me. Participant 14.

Occasional lapses in memory lead to experiences of disorientation and confusion. Participant 23 described the experience as a ‘full blank moment’ and as ‘disconcerting’.

#### Language

Impairments in language affected word finding, reading comprehension and writing. Participant 14 described making errors in writing and proofreading at work which they didn’t do before, “I would read it and think this is great letter and then actually there’d be, like, a word missing.” Issues with communication included difficulty following conversations or abruptly deviating from the topic of conversation. A feeling of ‘disconnection in the brain’, while trying to communicate, was described by participants:

I'm often looking for words when I'm speaking and I think of a word, but I can't find it, like I know what it means and I know what I want to say, but it’s like it’s just disappeared from my brain. Participant 17.

#### Attention

Participants experienced impaired attention, particularly divided attention (multitasking) and maintaining selective/sustained attention, and the impact of this on daily life. Participant 6 described difficulty talking while driving and “could [only] retrieve the information” once ‘*stopped at the traffic lights*’. Participant 17 described the frustration of losing their attention, “I used to be able to sit down and watch films and things. Now, I find myself sometimes flicking the TV over because I can't, like, concentrate on stuff.”

#### Executive functioning

Executive dysfunction included difficulties with planning, decision-making and mental flexibility. For example, Participant 11 found difficulty following ‘*a logical train of thought*’ and ‘*getting things done around the house*’. The term ‘*decision fatigue*’ was used by participants 12 and 13 to describe the exhaustion associated making a choice out of many options, such as selecting a programme on the TV.

#### Processing speed

Accounts of cognitive dysfunction included descriptions of the delay involved in completing tasks or retrieving information, suggesting participants were experiencing reduced processing speed.

When I'm doing patients notes have to go over them like up to 10 times maybe, word by word to make sure I've put the correct word. People used to comment how quick I could, like, type and do the notes. Now it’s taking me a lot longer. Participant 15.

#### Contributing factors

Fatigue, poor sleep and headaches were reported to exacerbate cognitive symptoms. Participant 4 explained that tiredness makes their brain feel ‘*jumbled*’. Other participants described continuous cognitive symptoms, independent of other factors.

### Theme 2: impact on physical function and psychological well-being

Over half of the participants (14/24) experienced negative impacts on their daily lives including employment, physical functioning and psychological well-being, as a direct result of their cognitive symptoms. Of these, 10 participants reported a decline in their perceived work performance or changes in employment or education due to issues with their memory or concentration. This included being unable to return to their pre-COVID-19 careers, reducing working hours or adapting roles due to the self-perceived inability to uphold the cognitive demands and high responsibility of their previous roles. Participants were concerned about when or if their cognitive abilities would return and this was associated with a loss of professional identity:

Throughout my career has involved writing and also proofreading and copy editing. And I had to give that up for two years, and I was so scared that my ability would never come back … I've always seen myself as someone who works with words. Participant 9.

I've actually changed jobs at work, changing jobs on Monday, because the job I do requires a lot of concentration and I was just making so many mistakes. I've kind of taken a sideways step. Participant 24.

More than half of the participants (14/24) described the challenges of carrying out activities of daily living (ADL) including cooking, driving and shopping, that they were previously proficient in or enjoyed:

The odd time I have actually forgotten to turn the cooker off as well. So I'm not, like, keen to cook a lot these days either, which I've always enjoyed cooking. Participant 17.

10 participants experienced psychological impacts as a result of their cognitive symptoms. Cognitive impairment contributed to low mood, stress and anxiety. Participant 16 described the experience of losing their memory abilities as ‘stressful’ and feeling ‘so down’. Participants reported concern about their cognitive health including experiencing a sense of accelerated ageing, and anxiety over whether these cognitive changes might signal the onset of age-related neurodegenerative diseases.

A few weeks into having Covid I felt like I'd aged 20 years and lost 20 IQ points….It makes you worried that there are other things, I think, oh my God have I actually got Alzheimer’s, you know? Because I could tick most of the boxes. Participant 12.

### Theme 3: symptom management

Half of the participants (12/24) reported strategies they had tried to manage their cognitive symptoms, with variable success. Participants described simplifying daily life by reducing demands or decision-making:

I find decisions tiring… So I've just eliminated that, I just wear the same coloured clothes every day [and] cook the same meals Participant 13.

Strategies for recording information and setting reminders were reported, including making to-do lists, setting alarms to take medication or remember dates and appointments. The effectiveness of these strategies for improving cognitive function was varied, with at least five participants reporting continued difficulties with memory and forgetfulness despite using strategies. Participant 19 attempted to use pill boxes and phone notifications to remind them to take their medication; however, they explained “I would still forget… I'll see the notification, and I think right, I'll do that in a minute and then I'll get distracted and I'll forget about it.”

Regular rest and breaks were required, particularly throughout the working day. Participant 4 took regular breaks while working from home, and explained “if I didn't do that, I won't be able to continue with my day.”

Several participants (7/24) described feelings of frustration and tediousness over the increased time or effort required to implement these strategies in order to complete tasks:

I just have to stop organising [meetings] any time after about 2 o'clock because I then I can't find the words that I'm trying to say and it sounds like ridiculous… That’s really, really frustrating Participant 23.

One participant attempted to regain their pre-COVID-19 cognitive function by challenging themselves in progressive increments, with some success:

I struggled to even just reading a page of a book… I've progressed again over kind over the last few months, really just by setting myself the kind of challenge or time to just read one chapter or a few pages of the book every day. Participant 14.

## Discussion

This mixed-methods study aimed to explore the extent and lived experiences of cognitive symptoms in LC. The majority of participants in this study reported a decline in cognitive ability and concern about this change. Cognitive symptoms were among the most common symptoms reported alongside low energy, immobility and breathlessness. The experiences of subjective cognitive impairments included difficulties with memory, attention, language, executive function and processing speed. Cognitive symptoms had profound negative effects on participants’ ability to work, complete ADL, their psychological well-being and self-image, in some cases for >2 years after the initial infection. Participants attempted to manage their cognitive symptoms using strategies to reduce decision-making and mental load, facilitate memory and improve energy levels. However, the strategies were described as tedious and frustrating to implement, with mixed effectiveness. By combining quantitative and qualitative methods, this study found that cognitive symptoms are among the most prevalent symptoms in LC with considerable negative impacts on the well-being, functioning and daily lives of people with LC.

The findings of this study are consistent with previous research on both objective measures of cognitive impairment^5 6^ and the lived experiences of LC.^17 18^ Our findings strongly align with those of Callan *et al*,^18^ showing the experiences of subjective cognitive impairments, altered self-identity and the challenges of managing ongoing symptoms. Similar to previous studies, we found cognitive impairment in LC impacted occupational status, including reducing working hours, changing job roles and loss of pre-COVID-19 employment.^25^ Additionally, participants reported difficulties in completing ADL, which reflects previous research highlighting the functional limitations caused by cognitive decline in LC.^26^ High rates of mood disorders, such as anxiety and depression, were also prevalent among participants, reinforcing existing evidence that emotional and mental health challenges often accompany cognitive dysfunction in LC.^27^ Furthermore, many participants described living an altered lifestyle, consistent with prior findings that LC can affect individuals’ ability to engage in social, professional and personal activities.^28^ These results contribute to the growing literature documenting the far-reaching impact of ongoing cognitive impairment in LC. The novel findings of this study are the interactions found between the experiences of cognitive impairment, physical and psychological functioning and self-management of cognitive symptoms. Poor memory, attention and executive functioning affected many areas of daily functioning and contributed to a loss of self-worth and identity and reduced psychological well-being. Strategies used for self-management of cognitive symptoms involved living an altered lifestyle by reducing daily demands, activities, exercise and decision-making and implementing ‘reminders’ for tasks and regular rest, most of which added further distraction and frustration to daily life. This may reflect a vicious cycle of withdrawal from daily life, low mood and poor self-coping strategies that essentially exacerbate cognitive impairment.

**Figure 4:**
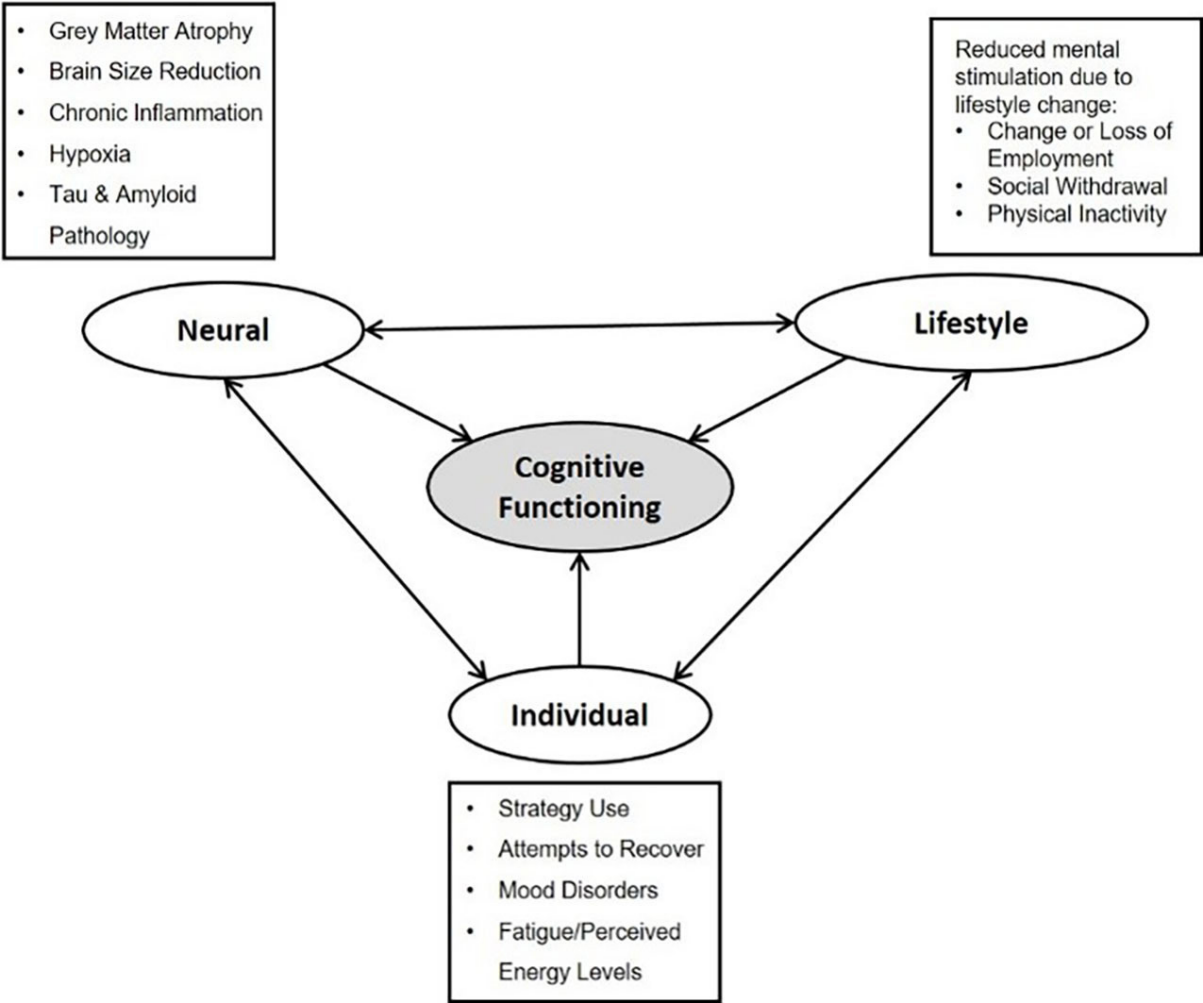
A conceptual framework of the Long-COVID Interacting Network of factors affecting Cognitive Symptoms.

Building on the findings of this study and prior research, we propose a new framework to conceptualise the factors contributing to cognitive impairment in LC, which appear to reflect an interacting symptom network.^29^ The Long-COVID Interacting Network of factors affecting Cognitive Symptoms (LINCS) framework integrates the findings of this study and previous research showing the neural, lifestyle and individual factors shaping actual and self-perceived cognitive dysfunction in LC (see figure 4). The possible interplay between psychological, physical and social factors, and the contribution of this to ongoing cognitive dysfunction following COVID-19, has previously been suggested by Callan *et al*.^18^ The need for this framework stems from the lack of evidence-based recommendations for clinicians regarding LC treatment.^30^ Furthermore, patients with LC have reported the challenging experiences of accessing primary care such as receiving inconsistent advice from healthcare professionals, difficulty accessing and navigating services and a lack of treatment options.^31 32^ This model provides a comprehensive framework for understanding the complexity of cognitive dysfunction in LC patients, which emphasises the multifaceted nature of the condition and may inform a more holistic approach to treatment by considering biological mechanisms and internal and external factors. The aim of presenting the LINCS framework is to guide future research and clinical practice towards more personalised, integrated and multidisciplinary care strategies for managing cognitive symptoms in LC, in line with previously published recommendations.^31^,^33^

The LINCS framework also highlights neuroinflammatory processes and brain structural changes as primary biological drivers of cognitive impairment in LC. Previous reviews have reported on the current understanding of the neurobiology of LC.^12 34^ The COVID-19 infection triggers an inflammatory response in the respiratory system, which can spread to the central nervous system (CNS). Within the CNS, cytokines, chemokines and activated microglial cells disrupt various neural cell types. This disruption interferes with the maintenance of myelin, impairs neuroplasticity and hinders hippocampal neurogenesis. It can also initiate neurotoxic responses in astrocytes, leading to a breakdown in neural circuits and cognitive impairment.^13^ COVID-19 infection can trigger antineural autoantibodies and T cells to provoke an autoimmune response, potentially leading to autoimmune encephalitis and contributing to continued neural damage.^35^ In more severe cases of COVID-19, nervous system injury can also result from hypoxia, further aggravating neuroinflammation.^14 15^ Structural changes to the brain following COVID-19 infection include reduced global brain size and grey matter thickness in the orbitofrontal cortex and parahippocampal gyrus.^9^ Several studies have highlighted the risks of developing abnormal tau pathology and cytotoxic amyloid protein aggregation in LC,^10 11^ which may indicate a future trajectory toward neurodegeneration.

Lifestyle factors also play a significant role in the cognitive impairment associated with LC. In a previous publication, we reported on the experiences and needs of individuals with LC and found that many participants experienced symptoms that significantly impacted their daily lives.^24^ Participants commonly reported challenges such as impaired physical mobility, cognitive dysfunction and social withdrawal. These difficulties often led to changes in employment, including reduced working hours, adjusting their role or being unable to return to work. Additionally, social withdrawal was frequently driven by a lack of understanding and support from family, friends and society, which contributed to feelings of shame and isolation. These changes in an individual’s engagement with their surroundings may reduce mental stimulation, further accelerating cognitive decline. Notably, both physical and social withdrawal, along with increased sedentary behaviour, are well-documented risk factors for the development of Dementia,^36 37^ highlighting the need to address these lifestyle factors when considering interventions for cognitive symptoms in LC patients.

The findings of the current study, in line with existing research, enhance our understanding of the individual factors that exacerbate cognitive symptoms in LC. Participants reported their efforts to recover by implementing coping strategies and managing fatigue and energy levels, while also dealing with the high prevalence of mood disorders. Prior evidence highlights the interconnectedness of these factors, suggesting they may collectively contribute to cognitive impairment in LC. For instance, fatigue and depression have been shown to impair cognition in conditions such as chronic fatigue syndrome and multiple sclerosis,^38 39^ and major depressive disorder is associated with reduced brain grey matter volume.^40^ Participants also described withdrawing from ADL as a way to manage symptoms. However, reduced cognitive, physical and social engagement, along with prolonged absence from work, have been associated with accelerated cognitive decline in mid to later adulthood.^41^,^43^ These findings emphasise the importance of addressing both the physical and psychological components in managing cognitive dysfunction in LC.

Given the evidence of accelerated cognitive ageing and dementia risk in LC,^9 10 16^ this clinical population of an estimated 2 million people in the UK^2^ may be the next generation at risk of developing age-related neurodegenerative diseases, fuelling a future epidemic of dementia in the UK. Currently, there is no approved treatment for LC,^44^ and the findings of this study highlight the vital need for an intervention to tackle the cognitive symptoms in LC, with aims to improve quality of life and reduce possible dementia risk. A recent scoping review evaluated treatments for cognitive impairment in LC and provided key recommendations for primary care management.^30^ The authors emphasised that a multidisciplinary, patient-centred approach is appropriate for addressing the cognitive symptoms. One of the main recommendations was for patients to adopt a healthy lifestyle, incorporating a balanced diet including key vitamins, micronutrients and probiotics, sufficient sleep, stress-reduction techniques, and appropriate physical activity. The WHO self-management booklet on LC provides a comprehensive strategy for returning to physical activity using a cautious pacing approach to mitigate overexertion.^33^ This approach contrasts with graded exercise therapy, which has been shown to potentially cause harm and worsen postexertional malaise in various chronic health conditions, including LC.^31^ Behavioural interventions were also identified, including cognitive training, mind–body interventions, music therapy and meditation.^30^ Overall, this scoping review underscored the importance of tailoring care plans to each individual, combining lifestyle modifications with medical and psychological interventions to create a comprehensive and adaptive treatment strategy for cognitive impairment.^30^ These recommendations align with the LINCS framework by advocating for a multidisciplinary and individualised approach to addressing the various factors which appear to exacerbate cognitive symptoms in LC. LC is a relatively new condition, and as such, the underlying pathology of cognitive impairment remains poorly understood and there is a current lack of high-quality evidence supporting targeted interventions for managing cognitive deficits in LC patients.^30^ Future research should prioritise identifying the precise mechanisms driving cognitive impairment in LC, including neuroinflammatory processes, neural dysregulation and neurovascular dysfunction.^34^ Additionally, there is a need for large-scale, high-quality, longitudinal studies to evaluate the effectiveness of both pharmacological and non-pharmacological interventions, such as cognitive rehabilitation and lifestyle modifications, in improving cognitive function. Developing standardised assessment tools and outcome measures will also be crucial to ensure consistency across studies and provide clearer guidance for clinical practice.^30^

The term ‘brain fog’ was used by some participants in this study to describe their cognitive symptoms, however previous evidence suggests the term lacks specificity and may not capture the severity of cognitive symptoms experienced in LC.^18^ The term ‘fog’ implies a perceptual deficit or ‘clouding’ of cognitive processes, whereas in this study, participants reported reduced processing speed, increased cognitive effort and a feeling of disconnection in the brain. Without the development of appropriate terminology to define these symptoms and a lack of recognition of the specific impairments to cognitive functioning, there is a barrier to medical diagnosis and the development of targeted cognitive interventions. An alternative code name has been proposed to describe the accelerated process of cognitive decline in pre-existing dementia following COVID-19, termed FADE-IN-MEMORY, that is, Fatigue, decreased Fluency, Attention deficit, Depression, Executive dysfunction, slowed INformation processing speed, and subcortical MEMORY impairment.^45^ The findings of this study show that FADE-IN-MEMORY might also be an appropriate term to describe the experience of cognitive symptoms in LC.

The strengths of this study include the rigorous and well-established methods to analyse the focus group data. The Framework Analysis^23^ is a data analysis technique that provides a structured yet flexible approach to processing qualitative data. By organising, coding, and conducting thematic analysis in a systematic manner, this method allows for efficient analysis of qualitative research while strengthening the credibility of the research process.^46^ The Framework Analysis is increasingly used in the analysis of qualitative data in multidisciplinary health research.^46^ The use of quantitative data collection to supplement the qualitative responses in this study allowed us to quantify the self-perceived change in cognitive abilities following LC and compare the severity of cognitive symptoms with other symptoms. However, the questionnaire rating scales were arbitrary, and the self-report nature of the questionnaire may not reflect an objective measure of cognitive functioning. Having said that, the extent of memory deficits following COVID-19 infection has been found to increase with the severity of self-reported ongoing symptoms,^7^ demonstrating the link between objective and subjective cognitive measures. One possibility for related future research would be to administer a brief test of global cognitive function, such as the Montreal Cognitive Assessment,^47^ to measure participants’ individual global cognitive function on the day of the focus group discussions. However, tests of global cognitive function primarily measure working memory and, therefore, may not capture the impairments in other cognitive domains participants reported in this study. There are also concerns that global screening tools, developed for identifying dementia and mild cognitive impairment, may lack sensitivity in reliability detecting cognitive deficits in LC.^48^ Furthermore, in this study, the self-report nature of the questionnaire complemented the qualitative data in which the overall aim was to investigate the lived experience of cognitive symptoms from the participants’ perspective, rather than to obtain an objective measurement of cognitive functioning. This study may be limited by the biased sample of mostly female and middle-aged participants, which prevented the comparison of possible age and gender differences in the experience of LC symptoms.

This mixed-methods study found that cognitive dysfunction was commonly reported by participants and had a significant impact on daily life and overall functioning. Participants were concerned about their cognitive decline and the limited options available for symptom management and treatment. There is a clear need for interventions that address the complex, multifactorial nature of cognitive impairment in LC. Without such targeted strategies, individuals with LC face an increased risk of accelerated cognitive ageing and the potential onset of dementia. This study introduced the LINCS framework and designed to provide a structured approach for clinicians to assess and manage the array of factors contributing to cognitive dysfunction in LC. By integrating multiple dimensions of cognitive health, the LINCS framework offers a valuable tool for both clinical practice and future research, which could be used to inform the development of interventions that are tailored to the needs of LC patients. Future high-quality, large-scale intervention studies are essential to identify effective treatments for cognitive dysfunction in individuals affected by LC.

## supplementary material

10.1136/bmjopen-2024-084999 online supplemental file 1

## Data Availability

Data are available on reasonable request.
