# Supplementary figures and images for: Exploring the experiences of cognitive symptoms in Long COVID: a mixed-methods study in the UK
Source: BMJ Open. 2025 Jan 25;15(1):e084999. doi: 10.1136/bmjopen-2024-084999 (PMC11784330; doi:10.1136/bmjopen-2024-084999)

**Supplementary Information**


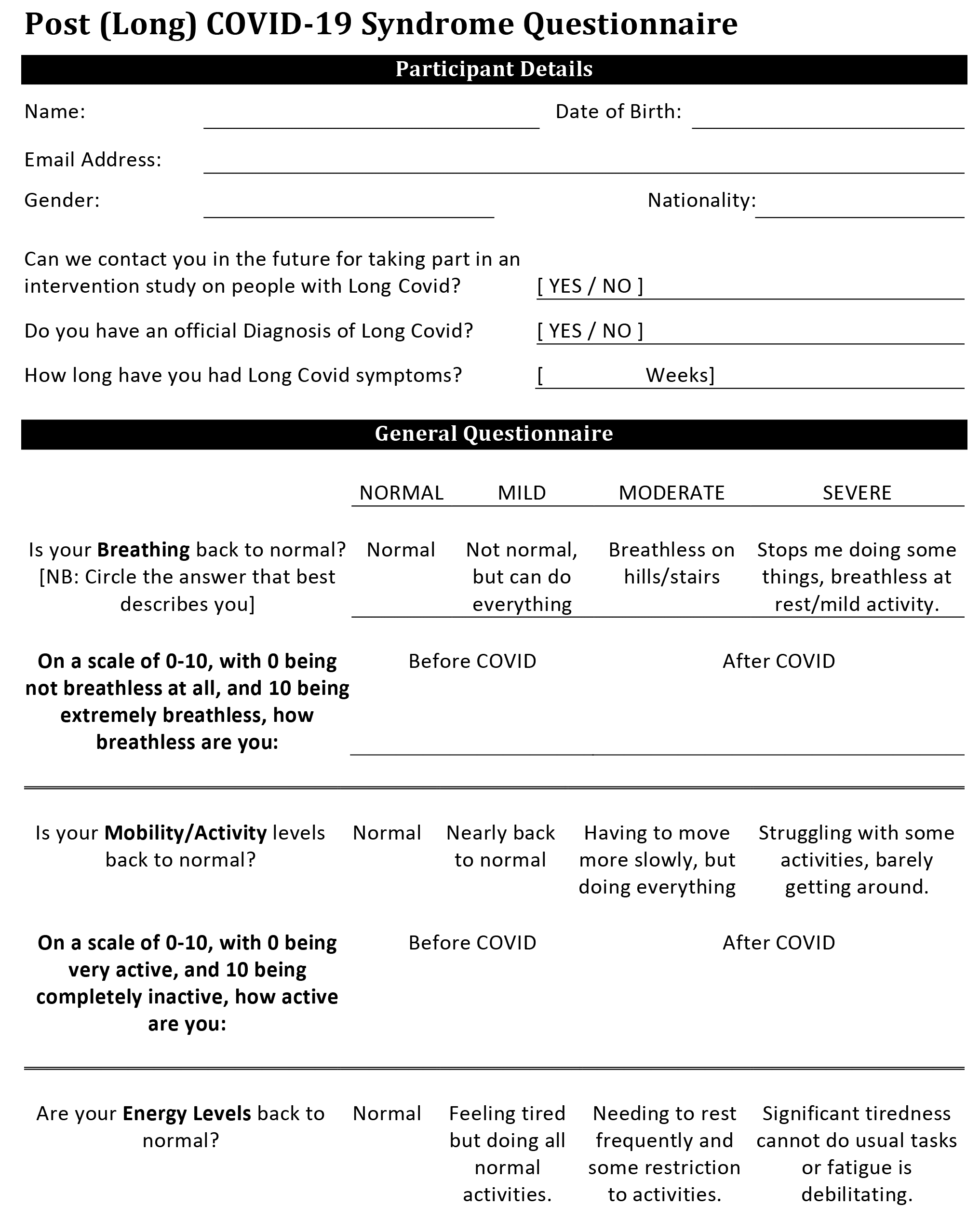
Participant Questionnaire


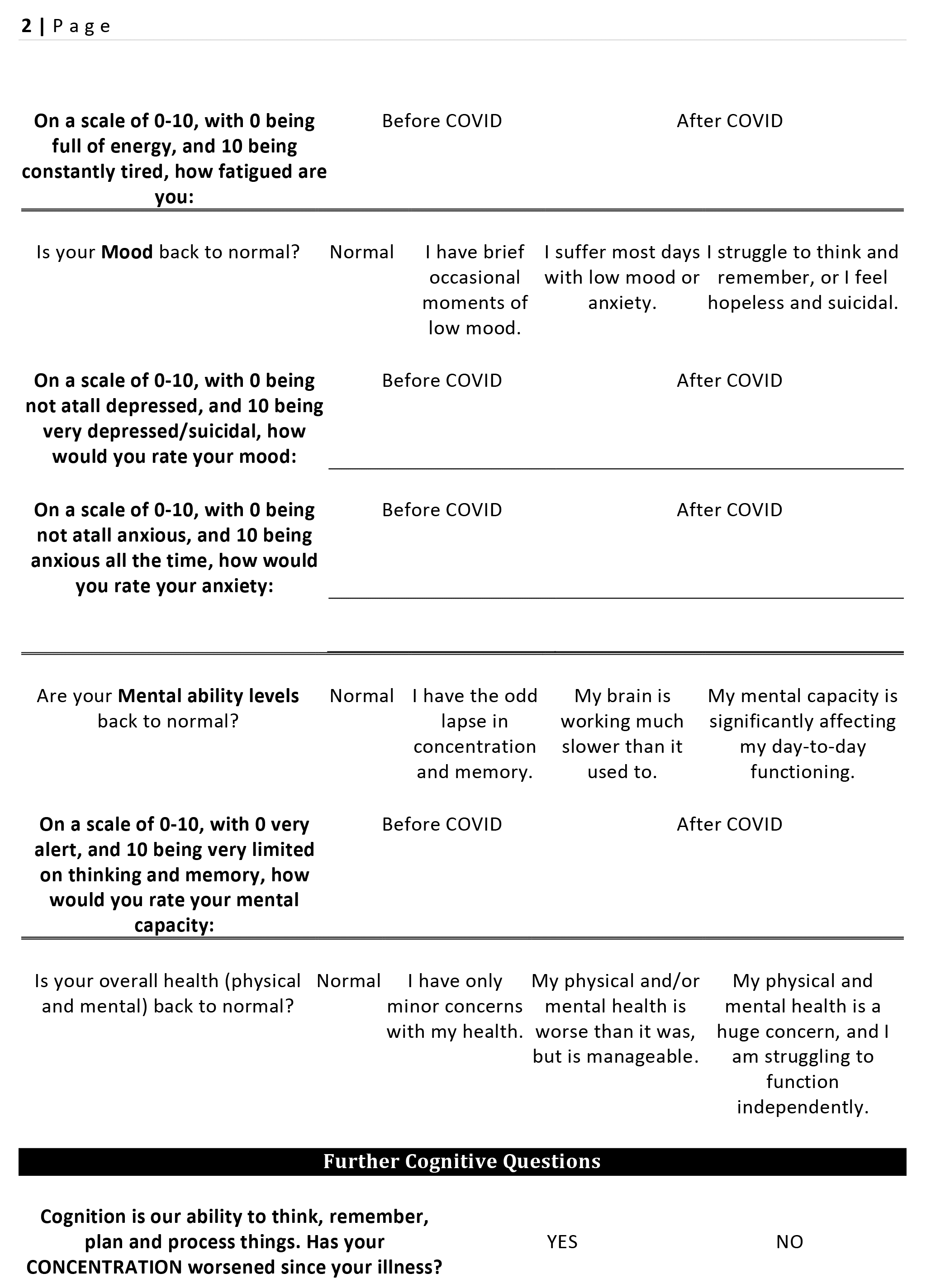


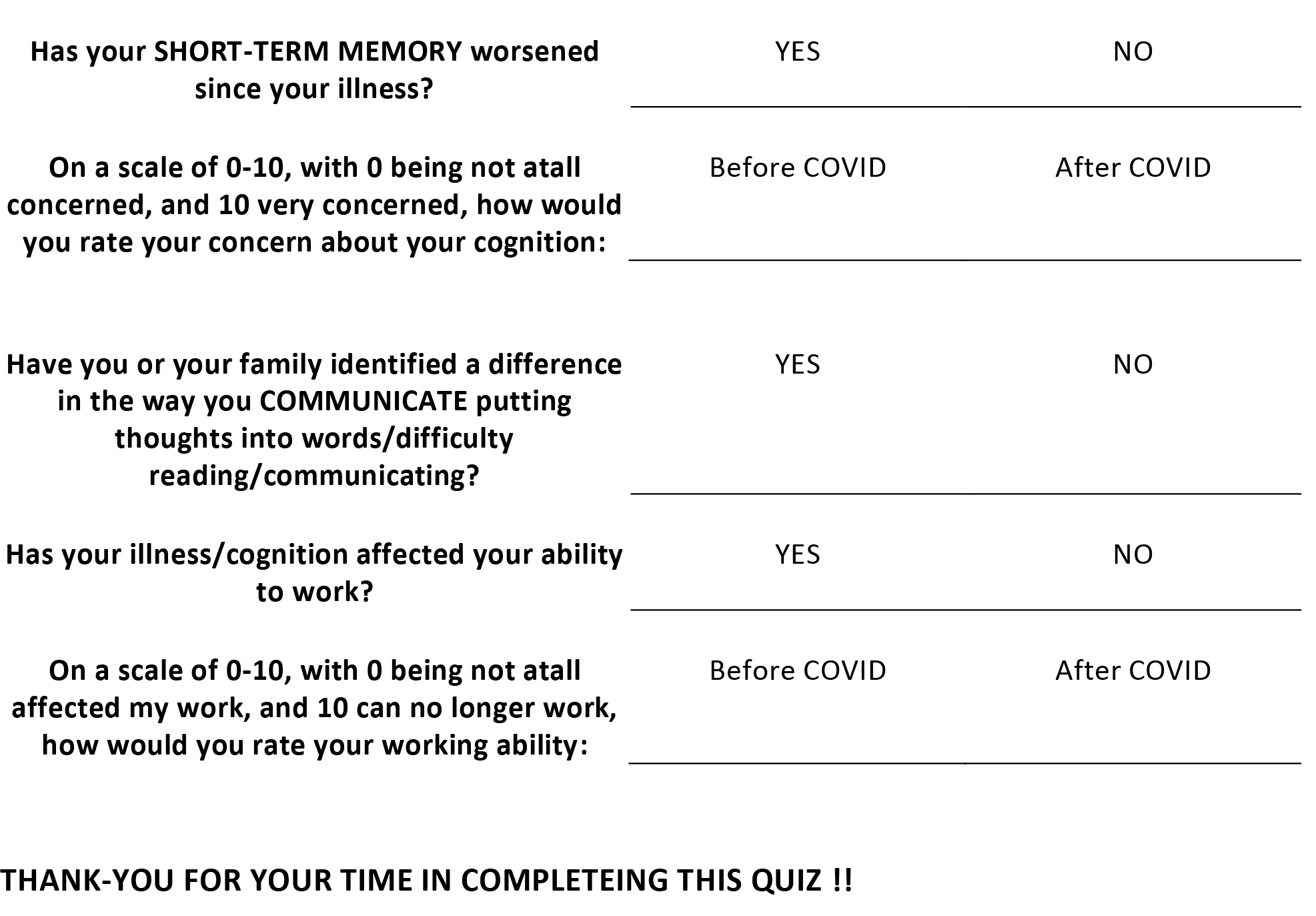

Supplement: online supplemental file 1 [file bmjopen-15-1-s001.docx]
