# Supplementary material for: Exploring the experiences of cognitive symptoms in Long COVID: a mixed-methods study in the UK
Source: BMJ Open. 2025 Jan 25;15(1):e084999. doi: 10.1136/bmjopen-2024-084999 (PMC11784330; doi:10.1136/bmjopen-2024-084999)
Supplement: Supplementary file 2 [file bmjopen-2024-084999.reviewer_comments.pdf]

PEER REVIEW HISTORY

BMJ Open publishes all reviews undertaken for accepted manuscripts. Reviewers are asked to complete a checklist review form and are provided with free text boxes to elaborate on their assessment. These free text comments are reproduced below.

ARTICLE DETAILS

Title (Provisional)

Exploring the experiences of cognitive symptoms in Long COVID: a mixed-methods study in the UK

Authors

Miller, Amy; Song, Ning; Sivan, Manoj; Chowdhury, Rumana; Burke, Melanie Rose

---

VERSION 1 - REVIEW

**Reviewer**	**1**
**Name**	**Cheke, Lucy G**
**Affiliation**	**University of Cambridge**
**Date**	**19-Mar-2024**
**COI**	**None.**

This paper covers a very important topic regarding the impact of cognitive dysfunction on the lives of those living with Long Covid. This type of in depth qualitative exploration can be extremely useful in helping researchers and clinicians better understand the experiences of patients and the impact on their lives.

However, I feel that the paper as currently presented falls short of doing that. Relatively major claims are made on the basis of the reported results, which are presented vaguely. Specifically, its very unclear how well supported by the participants each of the statements or themes identified are, as regular use of “some” and “others” accompany a single or pair of quotes, making it possible that all 25 participants reported something similar, or that only 1 or 2 did. It would be useful to have a proper thematic analysis where there’s some quantification of how often certain themes arise. For example, the fact the strategies were tedious and ineffective is mentioned several times in the summaries, but its hard to discern from the reported results how representative that was of the whole sample – how many had strategies, and of those how many thought them ineffective?

The authors present a conceptual model which covers the neural, environmental and individual factors that may contribute to cognitive dysfunction. However its not clear that the data that they present is sufficient to support the proposal of a new model, or that this suggestion adds anything to existing knowledge. However I can see that for clinicians making these links explicit in a conceptual model might be helpful. As such perhaps this can be justified by reference to the clinical need for better tools to assist primary care physicians with helping those with long Covid. There is certainly evidence that LC patients feel unable to access appropriate care. However there was no mention of clinical support in the results, despite 2 out of 6 of the guided questions being on that topic – why?

In the discussion the authors present some suggestions as to appropriate therapies based on things that have been found to lower dementia risk in (healthy) middle age. These suggestions are made without reference to any literature on LC therapies, and include "frequent and vigorous exercise". This section is irresponsible. There have been numerous studies on the efficacy of "graded exercise therapy" for LC all showing no effect or that its actively harmful due to the impact of post-exertional malaise – one of the most common symptoms of long covid. I advise to replace with a review of treatments that have been empirically explored or omit.

A more minor point, but overall the manuscript does not feel formatted as a final submission draft. The figures, heading/subheading etc. all feel like an earlier, unfinished draft. There are also inconsistencies in italicization, centering, several places with missing words.

Detailed comments

Introduction

- Could cite Guo et al 2022a and 2022b (Covcog1 and covcog2) – these discuss self reported cognitive symptoms and link them with objectively measured cognitive deficits. They also report on the impacts long covid have had on people's lives, including losing employment, struggling to get clinicians to take symptoms seriously, etc.

Methods

- Not sure we need a compete table of all participants in the main text in long form

- I think maybe more information is needed in the main body text about the structure of the focus groups, for example the guided questions should be there in a table (since there aren't many). Perhaps replace the table of participants with a table of these? Given that two questions focus on clinical services, its notable these weren't mentioned at all in the analysis – did patients indicate that there weren't any? If so this is worth noting.

Results

- 25 seems like a very small sample for this level of analysis. What is the justification for this sample?

- Figures need work (to be of a journal standard)

- Is theme 2 impract *of* physical function or impact *on* physical function?

- I'm not an expert in qualitative analysis, so maybe this is a misguided question, but there are a few times when the text refers to "some participants" or "others" and then its very unclear whether this is something that was reported by a significant proportion of the group, or just 1 or 2.

Discussion.

- "By combining quantitative and qualitative methods, this study revealed that cognitive symptoms are among the most prevalent and severe symptoms in LC with significant negative impacts on the wellbeing, functioning and daily life of people with LC" While this study has the potential to add richness and insight into this, it is overstating that it "reveals" it – this has been shown multiple times, not least by some of the studies reviewed in the introduction (e.g. Davies et al).

- Line 51-53, "found *in* this study"

- Reference to learned helplessness should be better justified or omitted. – LH is usually characterised by the stressor being inescapable and the individual thus no longer even trying to help themselves. Withdrawal from daily activities is itself an "escape" from the stressor – while I can see the similarities, the link as currently presented doesn't add anything.

- Literature review needed for treatments suggestions (see overall comments).

Reviewer 2

Name Hossain, Md Mahbub

Affiliation University of Houston

Date 20-Jun-2024

COI **None.**

This is a well-structured and timely study on long COVID. I appreciate the efforts and contributions of the authors, and I believe that revisiting some areas of this manuscript and editing a few sections may help communicating this research better.

First, figures 1 and 2 should be labeled as health problems or symptoms, whereas someone may find energy or mental ability had increased in the participants following long COVID. Please use and present the terms/constructs meaningfully that may prevent any confusion for future readers.

In the methods section, steps of analyzing data should be elaborated. It is not clear how framework synthesis was considered appropriate for this study, compared to other qualitative synthesis methods. Also, the approach for combining codes into sub-themes and themes should be explained in a way that other researchers, having access to your primary data, can possible replicate and find similar themes. The step-by-step analytical approach and iterative processes should be explained.

Lastly, the idea of proposing the LINCS framework is interesting; however, it should be grounded on the evidence emerged from the study findings. A grounded theory approach is probably better for exploring relationships between core ideas and formulating theoretical frameworks. If that approach is not feasible at this stage, please conduct an extensive literature review to strengthen the evidence base that may support your framework. In both cases, the framework should be supported or informed by the study findings, otherwise, it may not have much relevance to the primary objectives of the study. This framework can be very useful but a stronger methodological approach to developing/modifying this may affect its credibility or utility. Also, please consider explaining how this framework can be used for future research and practice in case of managing long COVID.

VERSION 1 - AUTHOR RESPONSE

Reviewer: 1
Dr. Lucy G Cheke, University of Cambridge
Comments to the Author:
This paper covers a very important topic regarding the impact of cognitive dysfunction on the lives of those living with Long Covid. This type of in depth qualitative exploration can be extremely useful in helping researchers and clinicians better understand the experiences of patients and the impact on their lives.

However, I feel that the paper as currently presented falls short of doing that. Relatively major claims are made on the basis of the reported results, which are presented vaguely. Specifically, its very unclear how well supported by the participants each of the statements or themes identified are, as regular use of "some" and "others" accompany a single or pair of quotes, making it possible that all 25 participants reported something similar, or that only 1 or 2 did. It would be useful to have a proper thematic analysis where there's some quantification of how often certain themes arise. For example, the fact the strategies were tedious and ineffective is mentioned several times in the summaries, but its hard to discern from the reported results how representative that was of the whole sample – how many had strategies, and of those how many thought them ineffective?

6. Thank you for your valuable feedback and for highlighting the importance of this topic. We have addressed your concern about the clarity of themes by providing quantification of how many participants contributed to each theme to clarify the extent of support for our findings, rather than referring to "some " or "others". For example, we now specify how many participants reported finding the strategies tedious and ineffective, thereby offering a clearer representation of their prevalence within the sample. Please refer to these changes in the Results section of the revised manuscript.

The authors present a conceptual model which covers the neural, environmental and individual factors that may contribute to cognitive dysfunction. However its not clear that the data that they present is sufficient to support the proposal of a new model, or that this suggestion adds anything to existing knowledge. However I can see that for clinicians making these links explicit in a conceptual model might be helpful. As such perhaps this can be justified by reference to the clinical need for better tools to assist primary care physicians with helping those with long Covid. There is certainly evidence that LC patients feel unable to access appropriate care. However there was no mention of clinical support in the results, despite 2 out of 6 of the guided questions being on that topic – why?

7. Thank you for this thoughtful feedback. In response to your comments, we have made several revisions to the manuscript to address your concerns. The justification for the proposed conceptual model has been clarified by emphasising its clinical relevance, particularly for clinicians who often face challenges in managing LC. In the revised version, we outline the gap in available tools for clinicians and reference the growing evidence that many LC patients feel unable to access appropriate care. This underscores the need for a more explicit framework to assist healthcare providers in identifying and addressing the multifactorial contributors to cognitive dysfunction in LC. These revisions help to justify the model, as a practical tool to inform clinicians and researchers on the multitude of relevant factors. We have also more clearly specified how the findings of this study support the model, specifically the individual factors. Please find these changes in the Discussion from page 16 onwards.

Regarding the comment on clinical support, this was not mentioned in the results as this topic did not serve to answer the research questions. The guided questions were designed to be broad, allowing participants to discuss various aspects of their experiences with LC. Initially, all discussions were coded into a broader framework, but this was deemed too extensive to include in a single paper. As a result, we chose to concentrate on experiences of cognitive symptoms in this manuscript as this was commonly described by participants and we believed this would be a relevant contribution to the current literature. Our previously published paper (Miller et al., 2024, link to paper here) explored the 'needs' of individuals with long COVID, including the need for developments to clinical services. We have now described this in the Methods section to clarify this approach. Please refer to the following extracts in the revised document:

'The guided questions were intentionally broad to encourage participants to explore various aspects of their experiences with long COVID. We intended to refine the analysis from this broader discussion to focus on key topics of particular relevance and importance.' Page 8.

'The initial framework was developed from the broader discussions and from this framework we identified common codes which were relevant to the research question to develop into themes.' Page 8.

'Whilst this analysis focused on investigating the experiences of cognitive symptoms, we have also reported on the broader perceived 'needs' of people with LC in our previous publication of this study (see Miller et al., 2024).' Page 9.

Reference: Miller A, Song N, Sivan M, et al. Identifying the needs of people with long COVID: a qualitative study in the UK. BMJ Open 2024; 14:e082728. doi:10.1136/bmjopen-2023-082728

In the discussion the authors present some suggestions as to appropriate therapies based on things that have been found to lower dementia risk in (healthy) middle age. These suggestions are made without reference to any literature on LC therapies, and include "frequent and vigorous exercise". This section is irresponsible. There have been numerous studies on the efficacy of "graded exercise therapy" for LC all showing no effect or that its actively harmful due to the impact of post-exertional malaise – one of the most common symptoms of long covid. I advise to replace with a review of treatments that have been empirically explored or omit.

8. Thank you for this suggestion to enhance the Discussion with a review of current therapies. In the revised manuscript, we have explored relevant literature to support the treatment suggestions. Since this manuscript was submitted, a scoping review was

published (in April 2024) which evaluated the available treatments for cognitive impairment in long COVID focusing on adopting a healthy lifestyle, improving nutrition and cognitive training. This provides a solid foundation of evidence to support these suggestions. Furthermore, we appreciate the concern about how to include physical exercise in this section. This point has been clarified by outlining the harmful risks of graded exercise for long COVID, whilst including the suggestion in the literature for a paced approach to exercise, focusing on individuals' capability. These revisions can be found in the Discussion on pages 18 and 19.

A more minor point, but overall the manuscript does not feel formatted as a final submission draft. The figures, heading/subheading etc. all feel like an earlier, unfinished draft. There are also inconsistencies in italicization, centering, several places with missing words.

9. Author Response: Thank you for pointing out the formatting issues and inconsistencies in the manuscript. We have carefully reviewed the document and addressed these concerns in the revised version. The figures, headings, and subheadings have been reformatted to ensure consistency and alignment with journal guidelines. Additionally, the italicisation, centering, and any missing words have been corrected throughout the manuscript. We appreciate your attention to these details and believe the manuscript is now more polished and suitable for final submission.

Detailed comments

Introduction

- Could cite Guo et al 2022a and 2022b (Covcog1 and covcog2) – these discuss self reported cognitive symptoms and link them with objectively measured cognitive deficits. They also report on the impacts long covid have had on people's lives, including losing employment, struggling to get clinicians to take symptoms seriously, etc.

10. In the revised manuscript, we have cited Guo et al. 2022b (COVCOG2) in the Introduction as this study identifies a link between objective and self-report measures of cognitive impairment in long COVID:

'Neuropsychological assessments and subjective reports have shown cognitive impairments in memory, attention, executive function and verbal fluency following SARS-CoV-2 infection in comparison to individuals without infection (5, 6). Objectively measured memory deficits following COVID-19 infection, show an increase in severity in line with the severity of self-reported ongoing symptoms (7).' Page 5.

Methods

- Not sure we need a compete table of all participants in the main text in long form

- I think maybe more information is needed in the main body text about the structure of the focus groups, for example the guided questions should be there in

a table (since there aren't many). Perhaps replace the table of participants with a table of these? Given that two questions focus on clinical services, its notable these weren't mentioned at all in the analysis – did patients indicate that there weren't any? If so this is worth noting.
11. Author Response: Thank you for your suggestions. In response, we removed the table of participants and have included a new table in the main body of the text outlining the guided questions used during the focus groups, as recommended. We have also provided more details about the discussion groups to give a clearer context on the structure of the discussions:

'The focus group discussions were audio-recorded and lasted approximately 2 hours. Participants were informed that the discussions were recorded and that any information they gave was confidential and would be anonymised. Each group contained two to five participants and one of four facilitators who informally followed guidance questions to encourage discussions around the main symptoms and experiences (see Table 1). The guided questions were intentionally broad to encourage participants to explore various aspects of their experiences with long COVID. We intended to refine the analysis from this broader discussion to hone in on key topics of particular relevance and importance. The questions were used to guide the sessions whilst the content of the discussions was self-directed by the participants in the focus groups.' Pages 7 & 8.

We acknowledge that the analysis does not include discussions about clinical services. For an explanation of our decision to focus the analysis on experiences of cognitive symptoms, please see our response in point 7.

Results
- 25 seems like a very small sample for this level of analysis. What is the justification for this sample?
- Figures need work (to be of a journal standard)
- Is theme 2 impract *of* physical function or impact *on* physical function?
- I'm not an expert in qualitative analysis, so maybe this is a misguided question, but there are a few times when the text refers to "some participants" or "others" and then its very unclear whether this is something that was reported by a significant proportion of the group, or just 1 or 2.
12. Author Response: The final sample size included 24 participants, and whilst may seem like a small sample, we consider it to be appropriate for the study design. This study was predominantly a qualitative study with some quantitative analysis (i.e. a mixed design). The aim of the study was to explore the experiences from a sample of people with long COVID, rather than to present population-level effects. As such, the quantitative summaries were designed to provide demographic and contextual information about participants' main symptoms, rather than to quantify the prevalence or intensity of these symptoms across the broader population. Furthermore, it was determined that data from

24 participants with 8 focus groups was sufficient to answer the research questions as data saturation was reached, i.e. no new themes were emerging from the analysis. In qualitative research, sample size is typically determined by data saturation rather than by statistical power, as in quantitative research. Finally, there should be a balance between the quantity and quality of qualitative data. In our focus groups, we specifically chose to have a greater number of groups (N = 8) with a small number of participants per group (2 to 5) to facilitate deeper and more meaningful discussions, thereby enhancing the richness of the data collected. Including a greater number of participants may not automatically improve the quality of the data and could dilute the findings by obscuring important nuances or patterns due to excessive information. In the revised manuscript, we have clarified the justification for this sample size in the Methods as follows:

'Participants included 25 adults with LC. Eligibility criteria included individuals with self-reported LC who lived in the UK. One participant did not return the Post-Covid Questionnaire so were removed from the analysis. The final sample size of 24 participants was sufficient to reach data saturation, where no additional codes or insights emerged from further discussions, ensuring the themes were rich and fully addressed the research question.' Page 7.

'There were 8 groups each containing two to five participants and one of four facilitators. These groups sizes were selected as previous evidence has shown that a larger number of groups containing fewer participants can yield more information than fewer groups with more participants per group (24).' Page 7.

The reviewer has commented that the figures need work but have not specified how they should be edited. Any inconsistencies in the figure captions and italicisation have been corrected.

Theme 2 should be impact *on* physical function. This has been corrected in the document.

Please refer to point 6, where we address the use of terms such as "some" or "others" in reporting participant responses. We have improved this section by providing quantification of responses within each theme to offer clearer insight into the extent and frequency of the reported experiences.

Discussion.
- "By combining quantitative and qualitative methods, this study revealed that cognitive symptoms are among the most prevalent and severe symptoms in LC with significant negative impacts on the wellbeing, functioning and daily life of people with LC" While this study has the potential to add richness and insight into this, it is overstating that it "reveals" it – this has been shown multiple times, not

least by some of the studies reviewed in the introduction (e.g. Davies et al).
- Line 51-53, "found *in* this study"
13. Author Response: These points have been corrected in the revised manuscript. In the Discussion the word "reveals" has been changed to "found".
Line 51-53, has been corrected to "found in this study".

- Reference to learned helplessness should be better justified or omitted. – LH is usually characterised by the stressor being inescapable and the individual thus no longer even trying to help themselves. Withdrawal from daily activities is itself an "escape" from the stressor – while I can see the similarities, the link as currently presented doesn't add anything.
14. Author Response: Thank you for this clarification. We have decided to omit the reference to learned helplessness to avoid confusion. In line with this change, 'Helplessness and fear' have been removed from the LINCS model.

- Literature review needed for treatments suggestions (see overall comments).
15. Author Response: Please refer to our response in point 8 for the review of treatment suggestions.

Reviewer: 2
Dr. Md Mahbub Hossain, University of Houston
Comments to the Author:
This is a well-structured and timely study on long COVID. I appreciate the efforts and contributions of the authors, and I believe that revisiting some areas of this manuscript and editing a few sections may help communicating this research better.
16. Author Response: Thank you for this positive feedback. We have endeavoured to address the comments below to further strengthen the paper.

First, figures 1 and 2 should be labeled as health problems or symptoms, whereas someone may find energy or mental ability had increased in the participants following long COVID. Please use and present the terms/constructs meaningfully that may prevent any confusion for future readers.
17. Author Response: We have edited the figure labels in the revised document from 'health domains' to 'health symptoms' in figures 1 and 2 to avoid confusion.

In the methods section, steps of analyzing data should be elaborated. It is not clear how framework synthesis was considered appropriate for this study, compared to other qualitative synthesis methods. Also, the approach for combining codes into sub-themes and themes should be explained in a way that other researchers, having access to your primary data, can possible replicate and find similar themes. The step-by-step analytical approach and iterative processes should be explained.

18. Author Response: Thank you for this feedback. In response to your comment, we have revised the methods section to provide a step-by-step explanation of the data analysis process, as follows:

'The Framework Analysis involved four stages: (a) the process of data familiarisation involved repeatedly reading the transcripts and listening to the audio recordings, which allowed for the identification of initial codes; (b) data were 'open coded' and a framework was developed and refined within the research team; (c) manual coding was performed in NVivo (R14.23.1, QSR International) to index the data according to the framework. A second analyst independently double-coded 25% of the data (2/8 transcripts) to assess the quality of the coding and to enhance the reliability and validity of the data analysis. Any differences in the coding were resolved by consensus and the framework was altered accordingly. Then the framework was applied to all remaining transcripts. To ensure consistency in the coding and reduce possible bias, the second analyst (S.M.) checked 20% of the coding. Data saturation was reached when no new codes were emerging from the analysis… (d) Theme development involved mapping and interpreting codes, identifying relationships between them, and systematically grouping them into sub-themes. These sub-themes were then further refined and categorised into overarching themes that captured the key patterns and insights from the data.' Page 9.

We have clarified the rationale for choosing the Framework Analysis over other qualitative methods, emphasising its categorising data in a structured and systematic way, allowing for the development of new themes relevant to the study's objectives:

'The data was analysed according to the Framework Analysis proposed by Ritchie and Spencer (1994). The Framework Analysis provides a structured yet flexible approach to analysing qualitative data, facilitating the systematic organisation of large datasets and diverse participant experiences into themes. The Framework Analysis was selected over other qualitative methods, such as Grounded Theory and Interpretative Phenomenological Analysis (IPA), as the focus of this study was not to generate new theory or deeply analyse lived experiences, but to explore and categorise experiences within a broader thematic framework.' Page 9.

Additionally, we have elaborated on the process of combining codes into sub-themes and themes, outlining the steps taken to ensure a transparent and replicable process. This includes a description of how initial codes were generated, refined through team discussions, and systematically grouped into sub-themes and overarching themes. We believe these revisions provide a clearer understanding of the analytical approach, making it easier for other researchers to follow and potentially replicate the process with similar results.
'(d) Theme development involved mapping and interpreting codes, identifying relationships between them, and systematically grouping them into sub-themes. These

sub-themes were then further refined and categorised into overarching themes that captured the key patterns and insights from the data' Page 9.

Lastly, the idea of proposing the LINCS framework is interesting; however, it should be grounded on the evidence emerged from the study findings. A grounded theory approach is probably better for exploring relationships between core ideas and formulating theoretical frameworks. If that approach is not feasible at this stage, please conduct an extensive literature review to strengthen the evidence base that may support your framework. In both cases, the framework should be supported or informed by the study findings, otherwise, it may not have much relevance to the primary objectives of the study. This framework can be very useful but a stronger methodological approach to developing/modifying this may affect its credibility or utility. Also, please consider explaining how this framework can be used for future research and practice in case of managing long COVID.

19. Author Response: We appreciate that a grounded theory approach may be appropriate for exploring relationships between themes, however this level of analysis is beyond the scope this study. Developing a grounded theory would require a different methodological approach and a more in-depth exploration of relationships within the data, which was not the primary aim of this manuscript. We selected the Framework Analysis method due to the systematic and structured processes of coding the data and formulating themes.

In response to your suggestion, we have conducted a wider literature review to strengthen the evidence base supporting the framework. This expanded review draws on a broader range of studies related to cognitive dysfunction in LC, highlighting how existing research informs and supports the development of the framework. Additionally, we have made revisions to ensure that the description of the framework is clearly aligned with the study findings, thereby reinforcing its relevance to the primary objectives of the research. Specifically, the findings of this study contribute to the 'individual factors' in the model. Please refer to these changes in the Discussion, from page 16 onwards.

VERSION 2 - REVIEW

**Reviewer**	**2**
**Name**	**Hossain, Md Mahbub**
**Affiliation**	**University of Houston**
**Date**	**29-Nov-2024**
**COI**	

This revised version offers a much better understanding of the conditions explored. The authors have done a great job addressing many concerns raised earlier. However, there are a few issues that can be revisited to bring more clarity and scientific accuracy:

1. The title and objectives should specify the context of the study (i.e., the UK), as a small sample mixed-methods study cannot provide generalizable evidence for a larger geographic area or other populations affected by long COVID (LC).

2. The first objective of the study explores the "prevalence of cognitive symptoms in LC"- which can be inconsistent with the methodology from an epidemiological viewpoint. Although the authors reported a "data saturation" for limiting their sample size, a proper sample size calculation with appropriate statistical power would be necessary and the sample should have been drawn from a broader population if true "prevalence" has to be estimated. Interestingly, the early sections of the paper establish a ground to understand the extent/burden of LC, which can be done through a mixed-methods study. I would suggest the authors to make the wordings consistent with the rationale they presented and the methods they used, as those might not be feasible to revise, and a more appropriate framing of the objective (and relev might preserve the true value of this manuscript.

3. Both in the introduction and discussion, the authors may wish to explore the need for the study as well as the implications of the findings emphasizing health and social inequities associated with LC and related adverse experiences/outcomes. That would provide a stronger representation of what this study means for patients and communities, for both researchers and practitioners. Also, the authors may add recommendations that may advance health equity for LC patients in LC and globally grounded on their findings and insights generated during this research. Such tacit knowledge can be invaluable for the readers and users of this research.

4. The authors noted the no additional qualitative studies have been conducted after Callan et al. (in lines 15-19), whereas a growing number of studies suggest otherwise. The manuscript, both in the background and the interpretation of the results may benefit from the discourse presented in these recent studies:

a. Rofail D, Somersan-Karakaya S, Choi JY, et al. Thematic analysis to explore patients' experiences with long COVID-19: a conceptual model of symptoms and impacts on daily lives. BMJ open. 2024;14(1):e076992. doi:10.1136/bmjopen-2023-076992

b. Ignacio M, Oesterle S, Rodriguez-González N, et al. Limited Awareness of Long COVID Despite Common Experience of Symptoms Among African American/Black, Hispanic/Latino, and Indigenous Adults in Arizona. Journal of racial and ethnic health disparities. August 2024. doi:10.1007/s40615-024-02109-7

c. Schmachtenberg T, Müller F, Kranz J, et al. How do long COVID patients perceive their current life situation and occupational perspective? Results of a qualitative interview study in Germany. Frontiers in public health. 2023;11:1155193. doi:10.3389/fpubh.2023.1155193

d. Chasco EE, Dukes K, Jones D, Comellas AP, Hoffman RM, Garg A. Brain Fog and Fatigue following COVID-19 Infection: An Exploratory Study of Patient Experiences of Long COVID. International journal of environmental research and public health. 2022;19(23). doi:10.3390/ijerph192315499

VERSION 2 - AUTHOR RESPONSE

1. The title and objectives should specify the context of the study (i.e., the UK), as a small sample mixed-methods study cannot provide generalizable evidence for a larger geographic area or other populations affected by long COVID (LC).

1. Author response: We appreciate that the objectives could be more specific, so we have included the study context in the revised version: "To explore the lived experiences and extent of cognitive symptoms in long COVID (LC) in a UK-based sample".
The current title specifies the context of the study so this has not been changed: "Exploring the Experiences of Cognitive Symptoms in Long COVID: A Mixed-Methods Study in the UK".

2. The first objective of the study explores the "prevalence of cognitive symptoms in LC"- which can be inconsistent with the methodology from an epidemiological viewpoint. Although the authors reported a "data saturation" for limiting their sample size, a proper sample size calculation with appropriate statistical power would be necessary and the sample should have been drawn from a broader population if true "prevalence" has to be estimated. Interestingly, the early sections of the paper establish a ground to understand the extent/burden of LC, which can be done through a mixed-methods study. I would suggest the authors to make the wordings consistent with the rationale they presented and the methods they used, as those might not be feasible to revise, and a more appropriate framing of the objective (and relev might preserve the true value of this manuscript.

2. Author response: Thank you for this point. We recognise that the term "prevalence" could be misleading, and we have altered the wording of the research questions to "extent": "1) What is the extent of cognitive symptoms in LC? 2) What is the lived experience of cognitive symptoms in LC?"

3. Both in the introduction and discussion, the authors may wish to explore the need for the study as well as the implications of the findings emphasizing health and social inequities associated with LC and related adverse experiences/outcomes. That would provide a stronger representation of what this study means for patients and communities, for both researchers and practitioners. Also, the authors may add recommendations that may advance health equity for LC patients in LC and globally grounded on their findings and insights generated during this research. Such tacit knowledge can be invaluable for the readers and users of this research.

3. Author response: We acknowledge that health and social inequities are an important aspect of LC and appreciate the reviewer raising this issue. However, addressing these disparities is beyond the scope of this paper. The focus of this study was to explore participants' experiences of cognitive symptoms in LC, rather than examining inequalities in the care they received.
We agree that disparities in health and social care warrant a dedicated investigation. To this end, we have previously explored many of these issues in a separate publication: Miller et al. (2024), Identifying the needs of people with long COVID: a qualitative study in the UK, published in BMJ Open. On page 9, we have highlighted this paper for readers interested in the issues faced by people with LC and our

recommendations for policy and practice.

4. The authors noted the no additional qualitative studies have been conducted after Callan et al. (in lines 15-19), whereas a growing number of studies suggest otherwise. The manuscript, both in the background and the interpretation of the results may benefit from the discourse presented in these recent studies:
a. Rofail D, Somersan-Karakaya S, Choi JY, et al. Thematic analysis to explore patients' experiences with long COVID-19: a conceptual model of symptoms and impacts on daily lives. BMJ open. 2024;14(1):e076992. doi:10.1136/bmjopen-2023-076992
b. Ignacio M, Oesterle S, Rodriguez-González N, et al. Limited Awareness of Long COVID Despite Common Experience of Symptoms Among African American/Black, Hispanic/Latino, and Indigenous Adults in Arizona. Journal of racial and ethnic health disparities. August 2024. doi:10.1007/s40615-024-02109-7
c. Schmachtenberg T, Müller F, Kranz J, et al. How do long COVID patients perceive their current life situation and occupational perspective? Results of a qualitative interview study in Germany. Frontiers in public health. 2023;11:1155193. doi:10.3389/fpubh.2023.1155193
d. Chasco EE, Dukes K, Jones D, Comellas AP, Hoffman RM, Garg A. Brain Fog and Fatigue following COVID-19 Infection: An Exploratory Study of Patient Experiences of Long COVID. International journal of environmental research and public health. 2022;19(23). doi:10.3390/ijerph192315499

4. Author response: There appears to be a possible misunderstanding here. On page 6, in lines 15-19 we note that, "to our knowledge no further studies have directly investigated the qualitative experiences of cognitive symptoms in LC". Please note that we are specifically referring to studies exploring experiences of brain fog and altered cognitive function in LC, which is the focus of this paper. We are not referring to qualitative studies which have explored the 'general' experiences of LC, as the reviewer has listed here.
Qualitative studies exploring the experiences of cognitive symptoms/brain fog are limited in quantity, and there remains to be no new qualitative studies exploring this topic specifically, since Callan et al. (2021), which warrants the need for the current study.
